# Pulpal dressing condensation methods in pulpotomy for primary molars: An in-vitro comparative study

**DOI:** 10.1016/j.sdentj.2021.06.002

**Published:** 2021-06-06

**Authors:** Tarun Walia, Wed Fahmi Ali Alzayer, Mohammed Nemer A. Al Nemer

**Affiliations:** a Unit of Pediatric Dentistry, Department of Clinical Sciences, College of Dentistry, Ajman University, Ajman, United Arab Emirates; b Intern, Qatif central hospital, Al Qatif, Saudi Arabia; c Intern, College of Dentistry, Ajman University, Ajman, United Arab Emirates

**Keywords:** Pulpotomy, Pulpal base, Condensation, Primary molars

## Abstract

**Purpose:**

To compare three condensation techniques of zinc oxide eugenol (ZOE) as a pulpal dressing material during pulpotomy in extracted primary molars.

**Material and Methods:**

Sixty primary first and second molars were embedded in individual wax casts and divided into three groups consisting of 20 teeth each. In group I, the ZOE base was condensed by an amalgam condenser, while a moist cotton pellet was used in group II. A combination of an amalgam condenser and a wet cotton pellet tested the condensation mentod in group III. The condensation quality of the three techniques was evaluated through two digital periapical radiographs taken in a lateral and anterior-posterior direction.

**Results:**

Non-parametric Kruskal-Wallis test showed that there was no significant difference between the technique and quality of ZOE compaction. However, a significant difference was observed on condensation assessment for combined three groups on lateral radiographs vs the appearance on antero-posterior radiographs with the p-value set at < 0.05.

**Conclusion:**

Voids appeared with all three techniques. A combination of an amalgam condenser and the wet cotton wool pellet was the least effective method of condensation. Lateral radiographs revealed much fewer spaces between the ZOE and pulpal floor in comparison to antero-posterior images.

## 1 Introduction

The success rate of pulpotomy in primary dentition depends on several factors: (i) accurate diagnosis of pulpal status, (ii) complete pulp extirpation from the chamber (iii) adequate hemorrhage control, and (iv) effective long term extra coronal coverage (Holan et al., 2005). There is no doubt that the final restoration of pulpotomized teeth with the stainless steel crowns protects the coronal seal, however the tight cervical coverage of amputated pulp with a bacteria-proof material can also ensure the functionality of the tooth till its exfoliation. Placement of a sedative base in direct contact with the pulpal surface can play an important role in the healing process of the remnant pulp as its close approximation allows dressing properties to act effectively (Hilton, 2009).

Materials often used as pulpal base after coronal pulp extirpation include zinc oxide eugenol (ZOE), intermediate restorative material, calcium hydroxide and bio-inductive calcium silicate based materials such as mineral trioxide aggregate (MTA) and biodentine (Dhar et al., 2017, Maurizio et al., 2020). MTA pulpotmies are commonly recommended treatment option however, ZOE is still a frequently used dressing material with devitalization or preservation techniques due to its bactericidal, sedative action, and palliative properties (Gonzalez-Lara et al., 2016). The use of ZOE in an optimal concentration as a base may enhance pulpal healing (Hui-Derksen et al., 2013). Application of a base material on the pulpal floor can be done with different application methods such as amalgam condenser, cotton pellet (Atasever et al., 2019) or a combination of amalgam condenser and wet cotton wool pellet (Yildiz and Tosun, 2014). As per the author’s knowledge, no study has been conducted so far that assessed the efficacy of various condensation methods to compact pulpal dressing material in a pulpotomy procedure.

Conventional radiography with an intraoral periapical film results in the conversion of three-dimensional information of a structure into a two-dimension image. This can lead to a false positive assessment of pulpal dressing condensation. The good quality ZOE condensation in a mesio-distal dimension can blind its poor compaction in the opposite direction of antero-posterior orientation. The objective of this in-vitro study was to assess the condensation quality of the three techniques used to place ZOE on the pulpal floor as a pulpotomy base in extracted primary first and second molars on periapical radiographs taken in antero-posterior and lateral directions.

## 2 Materials and methods

Ethical clearance to conduct the study was obtained from the college research ethics committee.

### 2.1 Study design and sampling

The study was carried out on 60 extracted primary first and second molars (32 maxillary and 28 mandibular teeth). Included teeth were required to have enough structure of clinical crown to hold ZOE base, intact pulp chamber floor without any perforations and enough root length for the tooth to be embedded in the wax. Extracted teeth were distributed with systematic random sampling into three groups consisting of 20 teeth each with every third tooth allocated to the same group. In group I, ZOE was condensed by an amalgam condenser, while the moist cotton pellet was used to place the material in group II. Zinc oxide eugenol paste in group III was first compacted by an amalgam condenser and then pressed with a moist cotton pellet.

### 2.2 Setting up of extracted teeth into wax casts

Modeling set-up pink wax (Cavex, Haarlem, Netherlands) was heated and poured into a plastic x-ray model tray (Frasaco, Cologne, Germany). All selected teeth were mounted in the wax on a model tray as per their natural anatomical position. The trays were color-coded under their bases with a permanent marker in three different colors to highlight the method employed for ZOE condensation.

### 2.3 Pulpotomy procedure

Caries was removed from extracted teeth by a low-speed diamond round bur size #two, followed by access cavity preparation with #two and #three high-speed tungsten carbide burs. Deroofing of pulp chamber was carried out with a safe ended bur until clear straight-line access was obtained. Pulp chamber was thoroughly cleaned with a spoon excavator, washed with normal saline to remove all debris, and dried with an air spray.

### 2.4 ZOE dressing condensation in the pulp chamber

Zinc oxide eugenol (Dentonics, Charlotte, USA) paste was freshly mixed into a putty-like consistency, carried gently to the pulp chamber by plastic instrument and condensed with methods as assigned for the experimental groups. The thickness of the compacted bulk of material was maintained between 3 and 4 mm and it was ensured to cover all of the root canal orifices and the floor. Round non-serrated amalgam condenser with a diameter of 1.0 mm at the smaller end and handle no. 6 was used in groups I and III (Brasseler, Georgia, USA) to adapt mixed ZOE. In group I and first step of group III, ZOE cement was compacted pulpally and laterally with a condenser ensuring an effective seal by pressing it against the cavo-surface margins. In group II and second step of group III, the mixed material was pushed by an absorbent cotton pellet, size no 00 with a diameter of 4 mm (Roeko, Coltène/Whaledent, Ohio, USA). It was made wet by dipping in normal saline and the excess removed by thoroughly squeezing against the cotton wool roll. Care was taken to apply pressure in each canal orifice separately. The remaining pulpal space was filled with an intermediate restorative material (IRM, Dentsply**,** DeTrey GmbH, Konstanz, Germany).

### 2.5 Radiograph technique

**Fig. 1:**
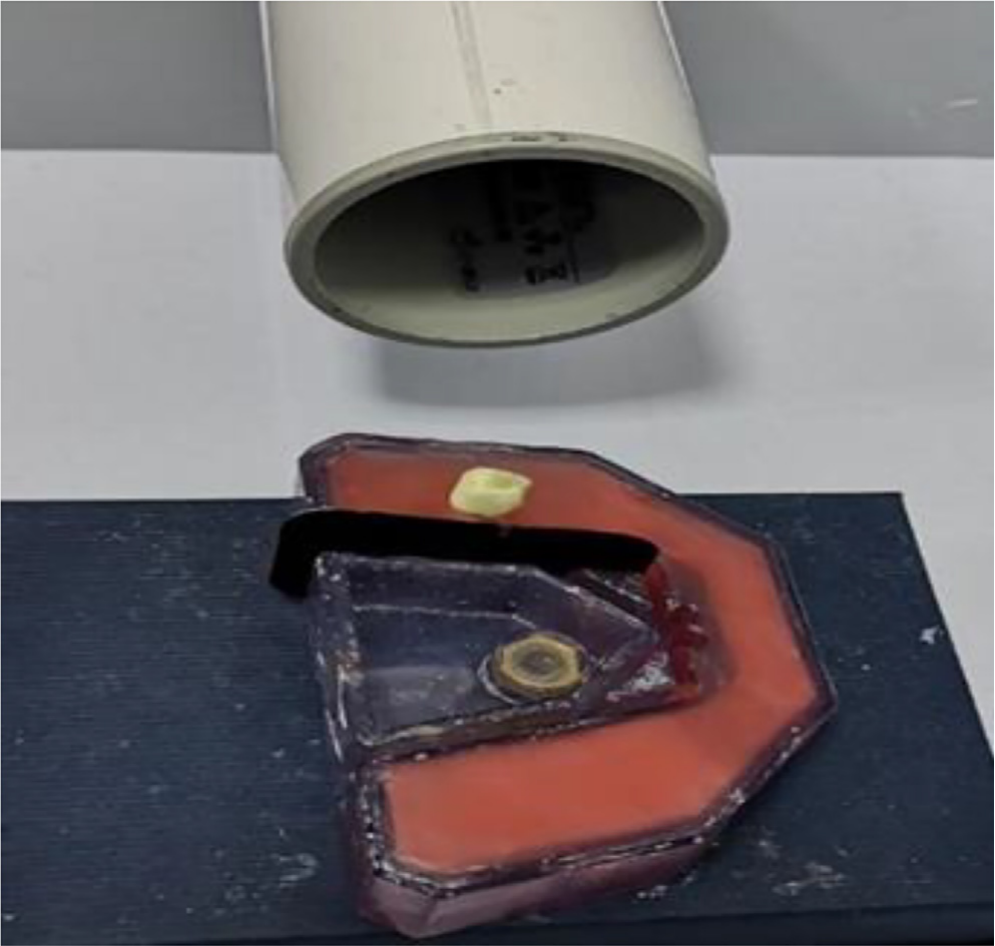
Radiographic technique with a mounted extracted tooth in wax cast.

Digital periapical radiographs were taken with 2.5 s of exposure time and 100 keV energy using an intraoral x-ray wireless unit (RXDC eXTend, MyRay, Imola, Italy). Standard adult x-ray films, size 0 with speed F (Kodak Insight, Eastman Kodak, Rochester, NY, USA) were utilized to take lateral and anterio-posterior (AP) radiographic images. The film was placed in the available slot on the x-ray model tray and two views were taken for each tooth with a long tube paralleling angle technique. As a consequence of the tooth being inserted in its natural anatomical position on the wax model tray, the first periapical radiograph taken in mesio-distal view accordingly evaluated ZOE condensation quality in the lateral direction (Fig. 1). Then the tooth was removed from the wax and embedded again in such a way that the newly mounted setting of the same tooth in the wax model tray was at a right angle (90°) to its previous position. This arrangement ensured that the second radiograph was taken in a bucco-lingual view which allowed assessment of ZOE condensation in the antero-posterior direction.

### 2.6 Standardization of pulpotomy and radiographic procedure

**Fig. 2:**
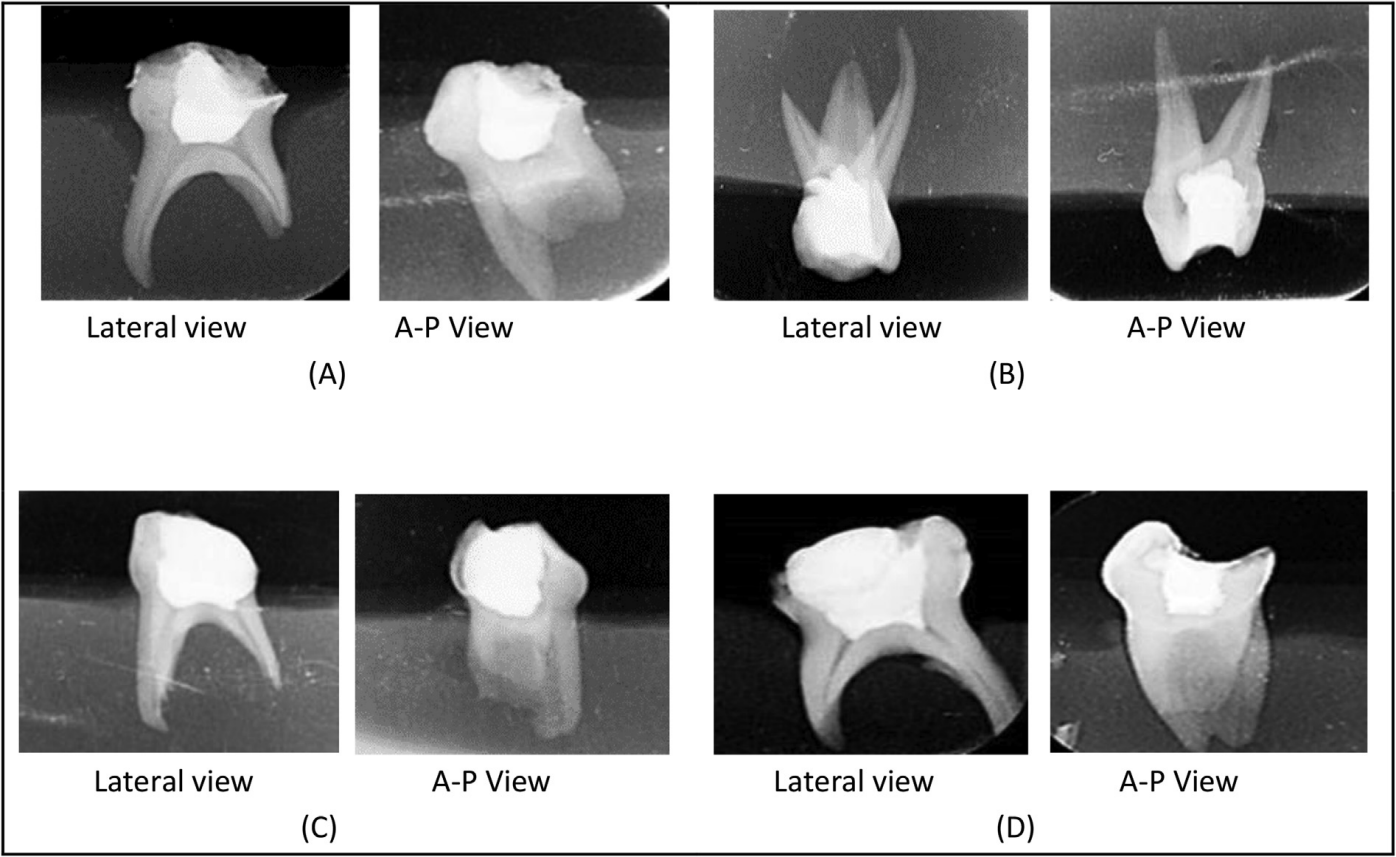
ZOE condensation initial scores (A) - Tooth with score 0; (B) - Tooth score 1; (C) Tooth with score 2 (D) Tooth with score 3.

Two operators were trained and calibrated through a pilot study on ten extracted teeth to standardize both procedures. Cohen’s κ was run to determine if there was an agreement between two observer’s inter-rater reliability for the pulpotomy and radiographic method. There was substantial agreement between two observer’s judgment, K = 0.761 (95% CI, 0.633 to 0.889), p < 0.05. The assessment of radiographic images was performed by a third evaluator (TW) who was blind to the techniques used for ZOE condensation. The radiographic images were sharpened with software (SCANORA 5.2.6, SOREDEX, Tuusula, Finland) zoom 3x and ZOE condensation was then assessed in two views according to the scoring system (Table 1) and (Fig. 2).

**Table 1:** Initial scoring system to assess the quality of ZOE condensation technique.

Initial Score	Description
0	Complete loss of contact between ZOE dressing and pulpal stumps in either AP or lateral view (Fig 2a)
1	Spaces between ZOE dressing and pulpal stumps in both AP or lateral view (Fig 2b)
2	Space between ZOE dressing and pulpal stumps in one direction while complete ZOE contact with pulpal stumps in the other view (Fig 2c)
3	Completely in contact with pulpal stumps in both AP and lateral direction (Fig 2d)

### 2.7 Calculation method for final adjusted score:

Criteria of the higher score were considered for calculation of the final adjusted score of a tooth if antero-posterior (AP) and lateral radiographic views had different scores. If a tooth scored one in AP image while the lateral radiograph score was zero for the same tooth, then the highest score of one was taken as a final score for that tooth. If the quality of ZOE condensation had an equal score both in AP and lateral views, then that score was considered as the final grade for the tooth

The final scores were entered in Statistical Package for Social Sciences 22 (IBM Corp., Armonk, NY, USA) and the data was analyzed quantitatively with a cross-tabulation method to find the relationship between the variables. Kruskal-Wallis test was used to determine the significant difference between three condensation techniques and radiographical methods. The statistical significance (p-Value) was set at below 0.05 with a 95% confidence interval.

## 3 Results

Fifty percent in group I teeth had complete compaction of the base material while in group II, where the ZOE was placed with a moist cotton pellet, the corresponding figure increased to 65%. In group III, the percentage of teeth with a final adjusted score of 3 was reduced to almost half of group II. Zinc oxide eugenol dressing was in close contact with the remaining pulp in only 30% of teeth which were filled with both amalgam condenser and moist cotton pellet (Table 2).

**Table 2:** Final adjusted scores of ZOE condensation in three groups.

	ZOE condensation method							
Final	Group I		Group II		Group III		Total	
Adjusted Scores*	Number %		Number %		Number %		Number %	
0	0	0.0	1	5.0	0	0.0	1	1.6
1	5	25.0	2	10.0	7	35.0	14	23.4
2	5	25.0	4	20.0	7	35.0	16	26.6
3	10	50.0	13	65.0	6	30.3	9	48.4
Total	20	100	20	100	20	100	60	100

*p-Value Final Adjusted Score − 0.11.

In Group II, the use of cotton pellets alone resulted in 30% teeth with the radiographic presence of gaps in either AP or lateral direction. However, the final adjusted scores of one and two increased to 50% and 70% of teeth respectively when amalgam condenser was used solely (group I) or in combination with moist cotton pellet (Group III).

Condensation quality was considered a failure if radiographic space was observed between ZOE paste and pulpal stumps in both bucco-lingual or mesio-distal views. Complete compaction of the dressing material in one or both radiographic directions was evaluated as success. This implied a final adjusted tooth score of zero or one was judged as inadequate ZOE condensation while the tooth with an adjusted score of two or three was considered to be an effective compaction method of the material. Group III had the highest failure rate of 35% with inadequate ZOE condensation while corresponding figures were 15% in group I and 25% in group II respectively. Successful placement of ZOE sub pulpal dressing was seen in 75% of teeth that were compacted with an amalgam condenser alone while 85% of teeth had an adequate condensation of ZOE material with only cotton pellet. In comparison, only 65% of teeth that were compacted with both amalgam condenser and cotton pellet had successful placement of the ZOE base. On intergroup comparative evaluation, there was no statistically significant difference between the three condensation techniques as the rate of effective condensation was high in all the experimental groups (p = 0.11).

Descriptive statistics revealed that complete compression of ZOE material over root canal orifices in two radiographic views was seen in only 49% in combined three experimental groups. Twenty-seven percent of all teeth in three groups had adequate placement of the material in either lateral or antero-posterior aspect while 24% of total teeth had some amount of space in both the radiographic dimensions resulting in deficient condensation of the dressing. When the quality of ZOE placement was evaluated on individual radiographic images, antero-posterior direction had a consistently higher number of scores of zero and one in all the three experimental groups in comparison to findings observed with lateral radiographic views. More than 55% of combined teeth in all groups had no contact or spaces were between ZOE material and pulp chamber walls when the condensation quality was seen on antero-posterior images. However, the corresponding figure reduced to only 33% on the conventional lateral periapical radiographs. The difference in the evaluation of ZOE condensation quality between two radiographic methods was non significant (Table 3).

**Table 3:** Radiographic evaluation of ZOE condensation quality in AP and lateral images.

Radiographic Direction	Score**	ZOE Condensation Method			Total	
		Group I (n)	Group II (n)	Group III (n)	Number %	
**Antero-posterior**	0	3	3	3	9	15.0
	1	8	7	9	24	40.0
	2	9	10	8	27	45.0
**p-Value**			0.88			
**Lateral**	0	0	1	0	1	1.6
	1	7	4	8	19	31.7
	2	13	15	12	40	66.7
**p-Value**			0.68			

*Score 3 was not considered for the individual x-ray evaluation as it is applicable only if the tooth scored 2 in both radiographic views.

## 4 Discussion

ZOE paste has been utilized as an effective pulp coverage material with ferric sulphate and laser-assisted pulpotomies due to its high success rate and antimicrobial activity (Hui-Derksen et al., 2013, Chien et al., 2001, Guelmann et al., 2002). Hume (1984) in his analytical study showed that the dentin adjacent to the ZOE filling contains bactericidal level of eugenol (10^-2^ mol/L) which acts as an anti-bacterial barrier to prevent microleakage of contaminated pulpal or oral fluids. However, the eugenol release reduces with time dramatically and the effectiveness of ZOE in excluding bacteria is reduced the longer it is in place in the mouth.

Condensation pressure is an uncontrolled variable in the placement of restorative dental materials that can affect the satisfactory seal against the cavo-surface margins (Nekoofar et al., 2017). Small condensers provide greater condensation force as condensation pressure is directly proportional to the diameter of a condenser (Wing, 1965). Condenser size in the present study had a diameter of 1.0 mm on the smaller end and 2.0 mm on the larger end so that the mixed material is adapted properly to all cavity walls, margins, and line angles. In addition to mechanical condensing tools, the wet cotton pellet can be used separately or in combination with hand condenser to place the pulpal medicament (Musale et al., 2018). Group II and the second step of group III in the current study, a smaller pellet size was applied to facilitate ZOE packing.

In the current study, interpretation of antero-posterior radiographs persistently revealed an inferior quality of ZOE compaction in all three experimental groups when compared to the outcome on lateral radiographs. Extrapolation of radiographic scores identified that antero-posterior images had 20% more teeth with either no contact or spaces between ZOE and pulpal walls than those discovered on lateral radiographs. This difference was statistically significant (p < 0.05) and can also be of clinical significance. The close approximation of ZOE material in the mesio-distal confines of the pulpal cavity must have blinded its inadequate condensation in the opposite bucco-lingual margins. Baccouche et al studied the topography of primary molars and observed that the greatest dimension of the pulp chamber was significantly higher in the buccolingual axis than mesiodistal particularly in primary maxillary molars (Baccouche et al., 2013). This difference in the pulp chamber proportions can contribute to a greater number of teeth with poor ZOE condensation as seen on antero-posterior xrays in the current study.

On intra-comparison of three techniques, group III produced the lowest number of teeth with adequate ZOE condensation. Since both condenser and the wet cotton wool pellet was used in group III, material pushed with condenser might have been displaced away from the internal cavo surface of the pulpal cavity by a cotton wool pellet. Group II showed the highest number of teeth with an effective seal of ZOE. The use of moistened cotton pellet reduced the attachment of the ZOE mixture unlike in group I where amalgam condenser alone was used. The adhesion of ZOE to the metal nip of the instrument can pull condensed material farther away from the internal walls of the pulp chamber. A requirement of a wide access opening during pulp chamber deroofing brings root canal orifices to the periphery and corners of the pulpal compartment (Rodd et al., 2016). The round contour of the condenser tip cannot push the material completely into margins of the triangular access cavity contours.

Only seventy-five percent of teeth in combined three groups had successful compaction of ZOE material on the pulpal floor. This is a significant finding as it implies that irrespective of the condensation method used, some voids can remain between the base medicament and the pulpal walls. These unfilled spaces in the pulp chamber can result in bacterial proliferation particularly if any subclinical inflammation already existed in the radicular pulp and the lack of tight seal will fail to preserve tissue vitality. Waterhouse et al., 2000, Levin, 1998 have stated that pulp needs to be protected from bacterial invasion and it will heal only in the absence of bacteria.

This study had limitations of smaller sample size and use of two -dimensional periapical radiographs. It would be meaningful to perform an in-vivo RCT to check if the quality of pulpal dressing has any effect on the overall clinical or radiographic success. Future studies can be planned to evaluate the efficacy of compaction with different tips of amalgam condenser; small increments vs bulk placement of pulpal base material.

## 5 Conclusion

(i) Perfect sealing of the coronal part of the canals with the ZOE base is not always possible to obtain in pulpotomy for primary molars.
(ii) A combination of amalgam condenser followed by wet cotton wool pellet was the least effective method of ZOE condensation.

## Declaration of Competing Interest

The authors declare that they have no known competing financial interests or personal relationships that could have appeared to influence the work reported in this paper.
